# Supplementary material for: Entamoeba histolytica: EhADH, an Alix Protein, Participates in Several Virulence Events through Its Different Domains
Source: Int J Mol Sci. 2024 Jul 11;25(14):7609. doi: 10.3390/ijms25147609 (PMC11277477; doi:10.3390/ijms25147609)
Supplement: Supplementary file 1 [file ijms-25-07609-s001.zip › ijms-3066127-supplementary material.pdf]

Supplementary Information

Materials and Methods

Viability assays

Trophozoites (0.2 × 10^5) were cultured in TYI-S medium supplemented with 3-5 mg/mL geneticin (G-418; Gibco) [34] and 1 µg/mL tetracycline (Sigma-Aldrich) to induce the expression of different EhADH domains and incubated at 37°C for several days. The number of trophozoites was counted every 24 h. Trophozoites viability was evaluated by trypan blue exclusion, using a 0.2% dye solution 687 and examined under the light microscope [17].

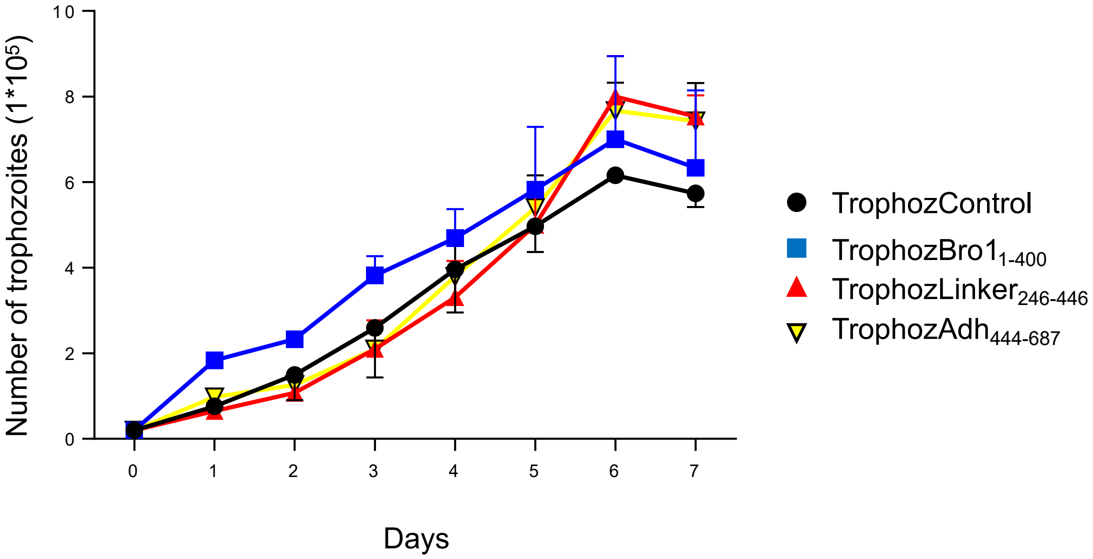

Figure S1. Cellular growth of transfected trophozoites. Viable parasites were counted during 7 days, using trypan blue exclusion test.

Table S1. Mass spectrometry analysis of *E. histolytica* proteins interacting with GST and His tags.

E. histolytica proteins interacting with GST tag			
**Accession number**	**Protein name**	**Functions**	**References**
EHI_142730	Actin, putative	Cytoskeleton	[27–29]
EHI_140120	Actin, putative	Cytoskeleton	[27–29]
EHI_009680	Small ribosomal subunit protein uS13	Ribosomal	[45]
EHI_011530	Ubiquitin-protein ligase, putative	Protein tag	[45]
EHI_078580	HECT-type E3 ubiquitin transferase	Unknown	[45]

E. histolytica proteins interacting with His tag			
**Accession number**	**Protein name**	**Functions**	**References**
EHI_163750	Actin, putative	Cytoskeleton	
EHI_107290	Actin, putative	Cytoskeleton	
EHI_159150	Actin, putative	Cytoskeleton	
EHI_126190	Actin, putative	Cytoskeleton	
EHI_043640	Actin, putative	Cytoskeleton	
EHI_182900	Actin	Cytoskeleton	
EHI_107290	Actin	Cytoskeleton	
EHI_031400	40S ribosomal protein S18, putative	Ribosomal	[45]
EHI_086120	40S ribosomal protein S18, putative	Ribosomal	[45]
EHI_009530	Pyruvate, phosphate dikinase	Enzyme	[56]
EHI_045090	Pyruvate phosphate dikinase AMP/ATP-binding domain-containing protein	Unknown	[56]
EHI_167320	Glyceraldehyde-3-phosphate dehydrogenase, putative	Enzyme	[45]
EHI_008200	Glyceraldehyde-3-phosphate dehydrogenase, putative	Enzyme	[45]
EHI_187020	Glyceraldehyde-3-phosphate dehydrogenase, putative	Enzyme	[45]

Table S2. Primers used to generate transfected trophozoites and recombinant proteins, embracing the EhADH domains

**Transfected trophozoites**		**Sequence primer (5′-3′)**	**Length (bp)**	**Restriction enzyme**	**Plasmid**	**Amplicon size (bp)**
**Bro1**	F	CCGGTACCTACCCATACGATGTTCCAGATTACGCTATGAATAGACAATTCATTCCTGAATT	61	*KpnI*	*pTet*	1221
	R	CCGGATCCTTAAATGTCTTGTGGAATATCACATG	34	*BamHI*		
**Linker**	F	CCGGTACCTACCCATACGATGTTCCAGATTACGCTATGACATTGGCTGCTAAAGGTGC	58	*KpnI*	*pTet*	631
	R	CCGGATCCTTATCTTCTTTCTTCAGCTGCTTCAG	34	*BamHI*		
**Adh**	F	CCGGTACCTACCCATACGATGTTCCAGATTACGCTATGGAAGCAGCTGAAGAAAGAAGATTAAG	64	*KpnI*	*pTet*	757
	R	CCGGATCCTTAAAGAGATGGAAACATAGGATTGG	34	*BamHI*		

**Recombinant proteins**		**Sequence primer (5′-3′)**	**Length (bp)**	**Restriction enzyme**	**Plasmid**	**Amplicon size (bp)**
GST-EhADH	F	CGGGATCCATGAATAGACAATTCATTCCT	29	*BamHI*	*pGEX-6P-1*	2077
	R	AGCTCGAGTTAAAGAGATGGA	21	*XhoI*		
His-Bro1	F	CGGGGTACCATGAATAGACAATTCATTCCTGAATT	35	*KpnI*	*pColdIDNA*	1221
	R	CCCGGATCCTAAAATGTCTTGTGGAATATCACATG	35	*BamHI*		
GST-Linker	F	CGCGTCGACGCATGACATTGGCTGCTAAAGGTGC	34	*SalI*	*pGEX-6P-1*	631
	R	GCCGCGGCCGCTTATCTTCTTTCTTCAGCTGCTTCAG	37	*NotI*		
GST-Adh	F	CGCGTCGACGCATGGAAGCAGCTGAAGAAAGAAGATTAAG	40	*SalI*	*pGEX-6P-1*	757
	R	GCCGCGGCCGCTTAAAGAGATGGAAACATAGGATTGG	37	*NotI*		

Table S3. Dataset assessed for normality from adhesion assays

Statistical test	TrophozControl	TrophozBro1$_{1-400}$	TrophozLinker$_{246-446}$	TrophozAdh$_{444-687}$
**Shapiro-Wilk test**	Normal	Normal	Normal	Normal
**W**	0.9817	0.9929	0.9877	0.9940
**Skewness**	0.1334	-0.3745	-0.3010	-0.02081
**Skewness shape**	Normal	Negative	Negative	Negative
**Kurtosis**	-1.866	-0.1334	0.9497	-1.098
**Kurtosis shape**	Platykurtic (negative)	Platykurtic (negative)	Platykurtic (negative)	Platykurtic (negative)
**Outliers**	0	0	0	0

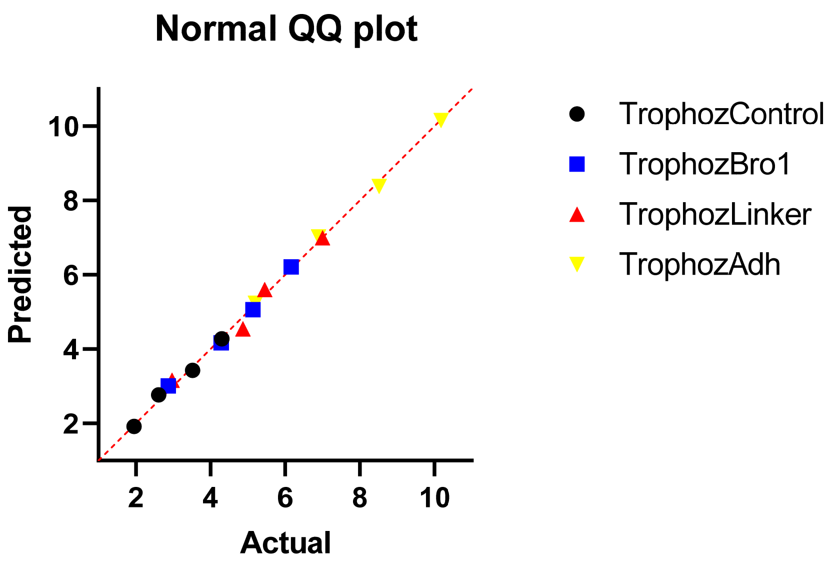

Figure S2. Normal QQplot of dataset from adhesion assays. Information was analyzed by GraphPad Prism 8.0 software.

Table S4. Dataset assessed for normality from erythrophagocytosis assays

Statistical test	TrophozControl	TrophozBro1$_{1-400}$	TrophozLinker$_{246-446}$	TrophozAdh$_{444-687}$
**Shapiro-Wilk test**	Normal	Normal	Normal	Normal
**W**	0.9969	0.9931	0.9873	0.9829
**Skewness**	0.1168	0.3638	0.1611	0.03959
**Skewness shape**	Normal	Normal	Normal	Normal
**Kurtosis**	-0.6925	0.4001	-1.477	-1.881
**Kurtosis shape**	Platykurtic (negative)	Platykurtic (negative)	Platykurtic (negative)	Platykurtic (negative)
**Outliers**	0	0	0	0

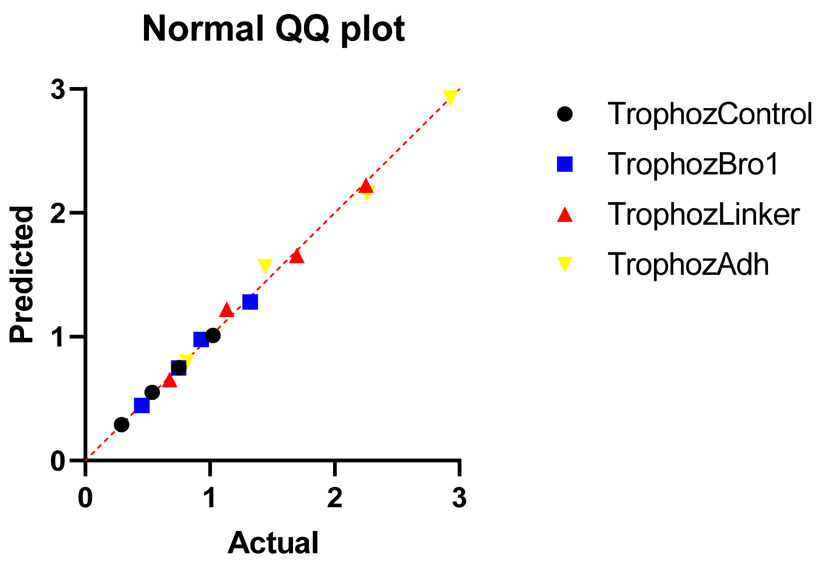

Figure S3. Normal QQplot of dataset from erythrophagocytosis assays. Information was analyzed by GraphPad Prism 8.0 software.

Table S5. Dataset assessed for normality from cytopathic assays

Statistical test	DMEM	TrophozControl	TrophozBro1$_{1-400}$	TrophozLinker$_{246-446}$	TrophozAdh$_{444-687}$
Shapiro-Wilk test		Normal	Normal	Normal	Normal
W		0.9233	0.9963	0.9811	0.9612
Skewness		1.292	0.3162	-0.6965	0.9709
Skewness shape		Positive	Normal	Negative	Normal
Kurtosis					
Kurtosis shape					
Outliers		0	0	0	0

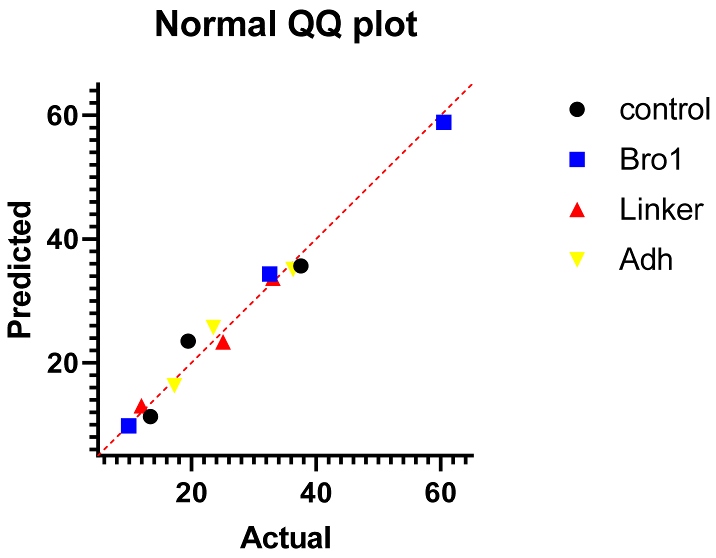

Figure S4. Normal QQplot of dataset from cytopathic assays. Information was analyzed by GraphPad Prism 8.0 software.

Table S6. Dataset assessed for normality from cytotoxic assays.

Statistical test	TrophozControl	TrophozBro1$_{1-400}$	TrophozLinker$_{246-446}$	TrophozAdh$_{444-687}$
**Shapiro-Wilk test**	Normal	Normal	Normal	Normal
**W**	0.9808	0.9995	0.9813	0.9733
**Skewness**	0.7009	0.1202	-0.6932	0.8184
**Skewness shape**	Normal	Normal	Negative	Normal
**Kurtosis**				
**Kurtosis shape**				
**Outliers**	0	0	0	0

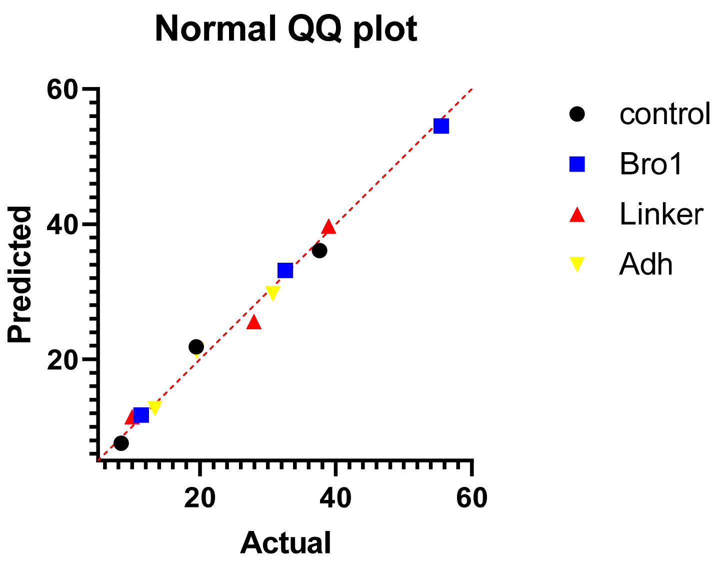

Figure S5. Normal QQplot of dataset from cytotoxic assays. Information was analyzed by GraphPad Prism 8.0 software.

Table S7. Dataset assessed for normality from TEER assays.

Statistical test	DMEM	TrophozControl	TrophozBro1$_{1-400}$	TrophozLinker$_{246-446}$	TrophozAdh$_{444-687}$
**Shapiro-Wilk test**	Normal	Normal	Normal	Normal	Normal
**W**	0.8626	0.7053	0.6491	0.8966	0.7451
**Skewness**	-0.3846	2.173	2.594	1.004	2.010
**Skewness shape**	Negative	Positive	Positive	Positive	Positive
**Kurtosis**	-1.825	4.811	7.190	0.04110	4.107
**Kurtosis shape**	Platykurtic (negative)	Leptokurtic (positive)	Leptokurtic (positive)	Platykurtic (negative)	Leptokurtic (positive)
**Outliers**	0	1	1	0	1

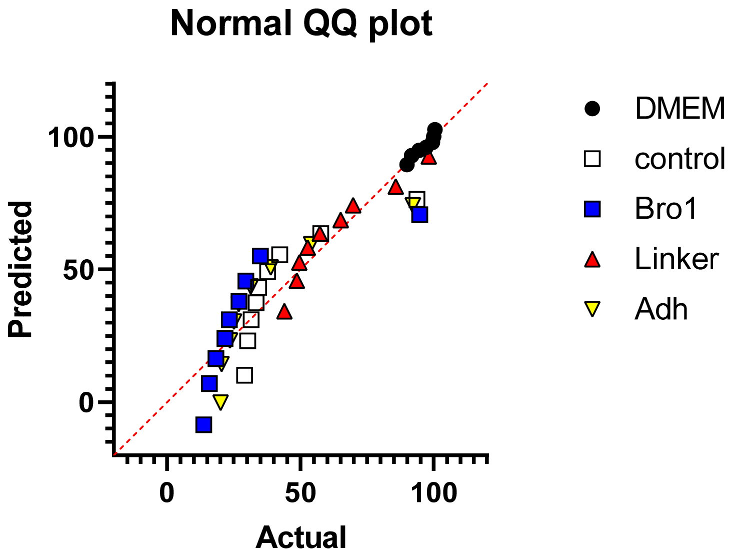

Figure S6. Normal QQplot of dataset from TEER assays. Information was analyzed by GraphPad Prism 8.0 software.

Table S8. Dataset assessed for normality from FITC-dextran assays.

Statistical test	EDTA	DMEM	TrophozControl	TrophozBro1$_{1-400}$	TrophozLinker$_{246-446}$	TrophozAdh$_{444-687}$
**Shapiro-Wilk test**		Normal	Normal	Normal	Normal	Normal
**W**	0.9600	0.9972	0.9847	0.9610	0.9532	0.9675
**Skewness**	0.4135	0.1633	0.3630	0.5003	0.8255	0.4359
**Skewness shape**	Normal	Normal	Normal	Normal	Normal	Normal
**Kurtosis**	-1.266	-0.3876	-0.7534	-1.133	0.03695	-1.185
**Kurtosis shape**	Platykurtic (negative)	Platykurtic (negative)	Platykurtic (negative)	Platykurtic (negative)	Platykurtic (negative)	Platykurtic (negative)
**Outliers**	0	0	0	0	0	0

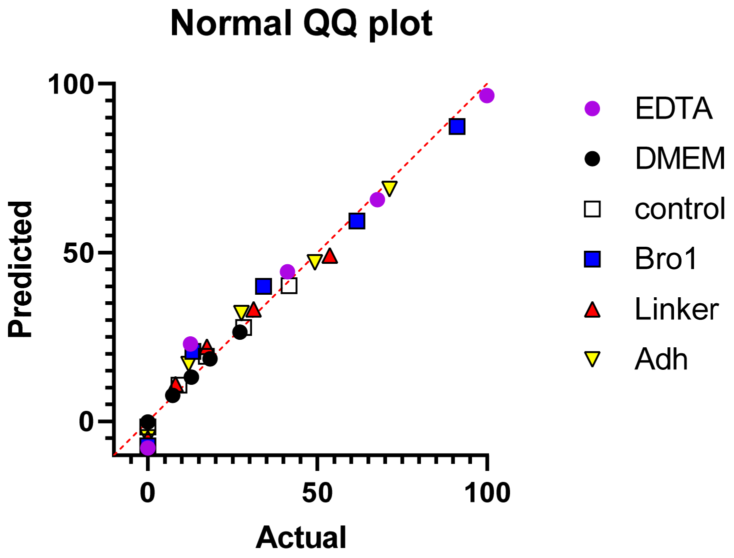

Figure S7. Normal QQplot of dataset from FITC-dextran assays. Information was analyzed by GraphPad Prism 8.0 software.

Table S9. Dataset assessed for normality from colon permeability assays.

Statistical test	PBS	TrophozControl	TrophozBro1$_{1-400}$	TrophozLinker$_{246-446}$	TrophozAdh$_{444-687}$
**Shapiro-Wilk test**	Normal	Normal	Normal	Normal	Normal
**W**					
**Skewness**					
**Skewness shape**	Normal	Normal	Normal	Normal	Normal
**Kurtosis**					
**Kurtosis shape**	Platykurtic (negative)	Platykurtic (negative)	Platykurtic (negative)	Platykurtic (negative)	Platykurtic (negative)
**Outliers**	0	0	0	0	0

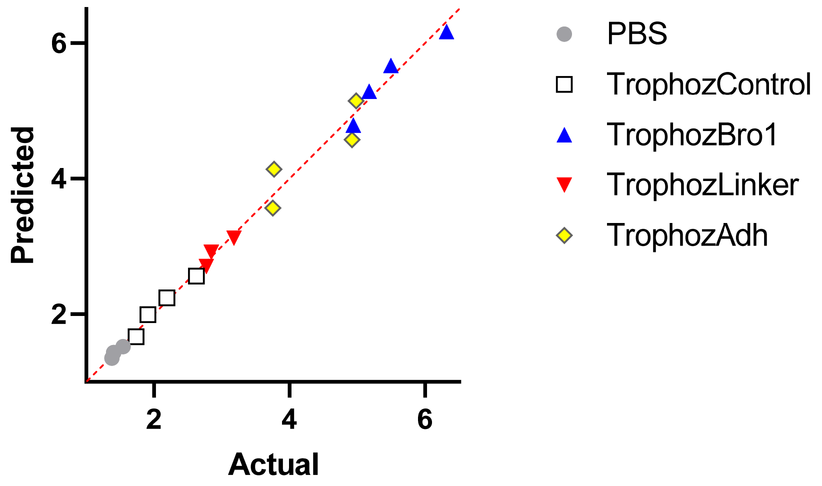

Figure S8. Normal QQplot of dataset from colon permeability assays. Information was analyzed by GraphPad Prism 8.0 software.

Table S10. Dataset assessed for normality from liver weight

Statistical test	Non infected	Infected
**Shapiro-Wilk test**	Normal	Normal
**W**	0.9379	0.9087
**Skewness**	-0.5551	-0.1222
**Skewness shape**	Negative	Negative
**Kurtosis**	-1.202	-4.272
**Kurtosis shape**	Platykurtic (negative)	Platykurtic (negative)
**Outliers**	0	0

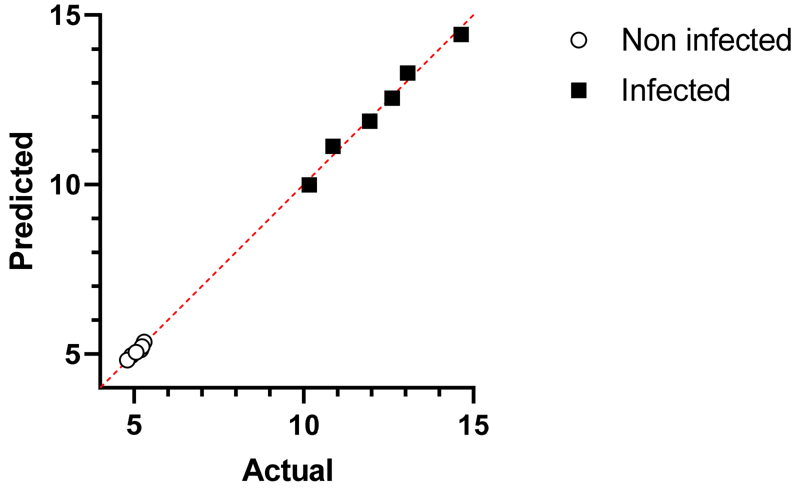

Figure S9. Normal QQ plot of dataset from colon liver weight. Information was analyzed by GraphPad Prism 8.0 software.

Table S11. Dataset assessed for normality from ALA formation assays

Statistical test	TrophozControl	TrophozBro1$_{1-400}$	TrophozLinker$_{246-446}$	TrophozAdh$_{444-687}$
**Shapiro-Wilk test**	Normal	Normal	Normal	Normal
**W**	0.9628	0.8796	0.9984	0.8947
**Skewness**	-0.6386	-1.270	0.000	1.331
**Skewness shape**	Negative	Negative	Normal	Positive
**Kurtosis**	1.500	2.426	0.3905	1.500
**Kurtosis shape**	Mesokurtic (normal)	Leptokurtic (positive)	Platykurtic (negative)	Mesokurtic (normal)
**Outliers**	0	0	0	0

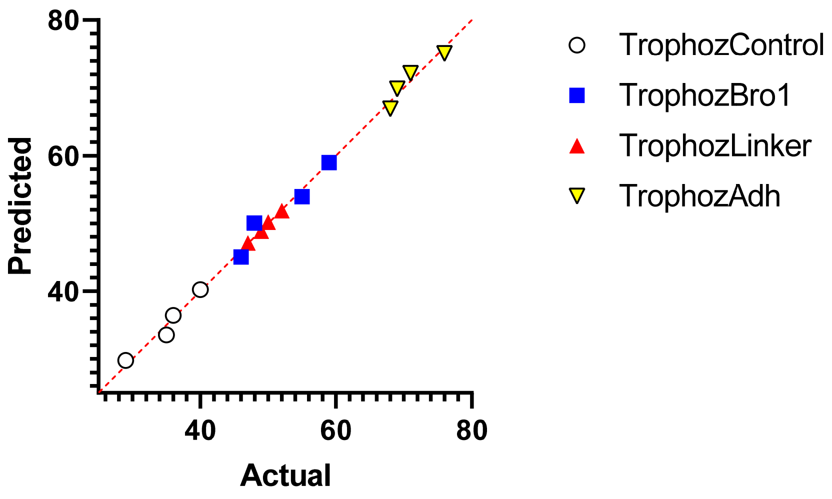

Figure S10. Normal QQ plot of dataset from ALA formation assays. Information was analyzed by GraphPad Prism 8.0 software.

References

14. Bañuelos, C.; García-Rivera, G.; López-Reyes, I.; Mendoza, L.; González-Robles, A.; Herranz, S.; Vincent, O.; Orozco, E. EhADH112 is a Bro1 domain-containing protein involved in the *Entamoeba histolytica* multivesicular bodies pathway. *J. Biomed. Biotechnol.* **2012**, *2012*, 657942. https://doi.org/10.1155/2012/657942.

27. Meza, I.; Sabanero, M.; Cazares, F.; Bryan, J. Isolation and characterization of actin from *Entamoeba histolytica*. *J. Biol. Chem.* **1983**, *258*, 3936–3941. https://doi.org/10.1016/s0021-9258(18)32757-1.

28. Manich, M.; Hernandez-Cuevas, N.; Ospina-Villa, J.D.; Syan, S.; Marchat, L.A.; Olivo-Marin, J.C.; Guillén, N. Morphodynamics of the actin-rich cytoskeleton in *Entamoeba histolytica*. *Front. Cell. Infect. Microbiol.* **2018**, *8*, 179. https://doi.org/10.3389/fcimb.2018.00179.

29. Rath, P.P.; Gourinath, S. The actin cytoskeleton orchestra in *Entamoeba histolytica*. *Proteins: Struct. Funct. Bioinform.* **2020**, *88*, 1361–1375. https://doi.org/10.1002/prot.25955.

32. Bosch, D.E.; Siderovski, D.P. G protein signaling in the parasite *Entamoeba histolytica*. *Exp. Mol. Med.* **2013**, *45*, e15. https://doi.org/10.1038/emm.2013.30.

33. Saito-Nakano, Y.; Loftus, B.J.; Hall, N.; Nozaki, T. The diversity of Rab GTPases in Entamoeba histolytica. *Exp. Parasitol.* **2005**, *110*, 244–252. https://doi.org/10.1016/j.exppara.2005.02.021

45. Dasrathy, S.; Sharma, M.P. Amebic liver abscess: A diagnostic challenge. *Trop. Gastroenterol.* **1995**, *16*, 1–2.

52. Verma, K.; Srivastava, V.K.; Datta, S. Rab GTPases take centre stage in understanding *Entamoeba histolytica* biology. *Small GTPases* **2020**, *11*, 320–333. https://doi.org/10.1080/21541248.2018.1528840

56. Rodríguez, M.A.; García-Pérez, R.M.; Mendoza, L.; Sánchez, T.; Guillen, N.; Orozco, E. The pyruvate:ferredoxin oxidoreductase enzyme is located in the plasma membrane and in a cytoplasmic structure in Entamoeba. *Microb. Pathog.* **1998**, *25*, 1–10. https://doi.org/10.1006/mpat.1998.0202
